# Mechanical Circulatory Support Devices in Patients with High-Risk Pulmonary Embolism

**DOI:** 10.3390/jcm13113161

**Published:** 2024-05-28

**Authors:** Rama Ellauzi, Saliha Erdem, Mohammad Fahad Salam, Ashish Kumar, Vikas Aggarwal, Gerald Koenig, Herbert D. Aronow, Mir Babar Basir

**Affiliations:** 1 Department of Internal Medicine, Henry Ford Hospital, Detroit, MI 48202, USA; 2 Department of Internal Medicine, Detroit Medical Center, Wayne State University, Detroit, MI 48202, USA; hj5215@wayne.edu; 3 Department of Internal Medicine, Michigan State University, East Lansing, MI 48502, USA; mohammadfahadsalam@hotmail.com; 4 Department of Internal Medicine, Cleveland Clinic Akron General, Akron, OH 44307, USA; kumarua@ccf.org; 5 Department of Cardiovascular Medicine, Henry Ford Hospital, Detroit, MI 48202, USA; vaggarw2@hfhs.org (V.A.); haronow1@hfhs.org (H.D.A.)

**Keywords:** massive pulmonary embolism, mechanical circulatory support device, right ventricular assist device

## Abstract

Pulmonary embolism (PE) is a common acute cardiovascular condition. Within this review, we discuss the incidence, pathophysiology, and treatment options for patients with high-risk and massive pulmonary embolisms. In particular, we focus on the role of mechanical circulatory support devices and their possible therapeutic benefits in patients who are unresponsive to standard therapeutic options. Moreover, attention is given to device selection criteria, weaning protocols, and complication mitigation strategies. Finally, we underscore the necessity for more comprehensive studies to corroborate the benefits and safety of MCS devices in PE management.

## 1. Introduction

Venous thromboembolism presenting as either deep vein thrombosis (DVT) or pulmonary embolism (PE) is the third most common acute cardiovascular condition, surpassed only by myocardial infarction and stroke [1]. In cases where standard therapeutic options are contraindicated or when pulmonary embolism (PE) remains unresponsive to medical interventions, mechanical circulatory support (MCS) devices may offer therapeutic benefit. However, the extent of scientific evidence evaluating the efficacy and outcomes of MCS devices in PE is sparse. In this review, we will critically analyze the diverse classifications of PE and explore current and emerging treatment options, emphasizing the role of MCS devices in treating patients with PE.

## 2. Defining High-Risk Pulmonary Embolism

PE is the obstruction of the pulmonary artery or its branches due to embolic material, most commonly thrombemboli. The development of PE follows a pathogenic process akin to that of thrombus formation, including venous stasis, endothelial injury, and a state of hypercoagulability [2]. Based on patient outcomes from acute PE, the American Heart Association (AHA) [3] and the European Society of Cardiology (ESC) [4] classify PE based on severity. ‘Massive’ PE per the AHA, or ‘high-risk’ PE per the ESC, is the most severe, characterized by hemodynamic instability, which is defined as persistent hypotension (systolic blood pressure <90 mm Hg for at least 15 min or requirement for inotropic support), pulselessness, or persistent profound bradycardia. These hemodynamic changes result from the PE and are not due to other factors such as arrhythmia, hypovolemia, sepsis, or left ventricular (LV) dysfunction [4,5].

## 3. Pathophysiology

**Figure 1:**
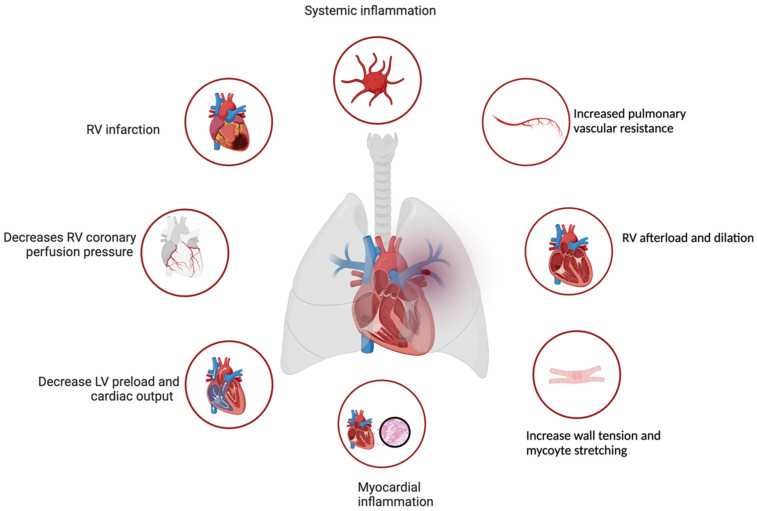
Pathophysiology of the changes that occur during a high-risk pulmonary embolism. RV, right ventricular; LV left ventricular (Created with BioRender.com).

It is critical to gauge PE severity at the time of diagnosis. High-risk PE implies hemodynamic instability, which can occur through multiple mechanisms, predominantly affecting right ventricular function. Increases in right ventricular (RV) afterload provoke RV dilation and extend RV contraction time due to increased wall tension and myocyte stretching. This, in turn, can induce a shift of the interventricular septum towards the left, adversely impacting LV preload and, ultimately, overall cardiac output [6,7]. Moreover, PE can amplify pulmonary vascular resistance, intensifying systemic inflammation, and thrombin and platelet activation, disrupting both circulation and gas exchange [8]. The consequent reduction in RV coronary perfusion pressure leads to increased RV end-diastolic pressure, coronary venous pressure, ventricular wall stress, and oxygen demand and the resultant coronary ischemia may trigger RV infarction and failure, further exacerbating hemodynamic instability, Figure 1 [6,7].

## 4. Incidence, Predictors of Mortality 

PE impacts nearly 900,000 individuals annually in the United States (US) [9], resulting in an estimated 100,000 deaths each year [10]. Early hospitalization and 30-day mortality are driven by multiple factors, chief among them the presence of right ventricular dysfunction and/or hemodynamic instability [4,11,12,13,14]. The survival rate for patients with severe pulmonary embolism who require extracorporeal membrane oxygenation device (ECMO) ranges between 38% and 67% [15,16,17,18]. Cardiac arrest before ECMO cannulation is predictive of poorer outcomes [16,18]. Evidence supporting other MCS devices, such as Impella RP or Tandem Heart (Protek Duo), is limited and primarily based on case series [11,19,20]

## 5. Treatment

### 5.1. Pert Team

A pulmonary embolism response team (PERT) is a multidisciplinary team that involves experts from various medical specialties, which may include cardiovascular medicine, pulmonary critical care, hematology, vascular medicine, anaesthesiology, intensive care, cardiothoracic surgery, vascular surgery, interventional radiology and others. PERT teams aim to address the treatment and knowledge gaps inherent in caring for patients with PE by leveraging the team’s diverse expertise. Because level-one evidence for MCS in this context is lacking, clinicians have historically relied on individual judgment to assess the safety and effectiveness of these devices when used to treat PE. The PERT model convenes specialists from diverse backgrounds to collaboratively determine more informed and balanced, consensus-driven treatment decisions [5]. Multi-specialty guidelines encourage PERT teams in the setting of high- and intermediate high-risk PE. This approach has had a favorable impact on coordination between specialties and treatment decision-making. Notably, PERT-initiated care versus standard of care has been associated with reduced 12-month mortality in patients with high- and intermediate high-risk PE [21]. In PERT team discussions, consideration should be given to the need for mechanical circulatory support.

### 5.2. Anticoagulation

In acute high-risk PE, immediate administration of anticoagulation is recommended in those without absolute contraindications to anticoagulation therapy, such as hemorrhagic stroke, recent major surgery, and bleeding diatheses. The use of subcutaneous weight-adjusted low-molecular-weight heparin, fondaparinuax, unfractionated heparin and non-vitamin K anticoagulants (NOACs) can be considered [4]. Intravenous unfractionated heparin is favored in patients with hemodynamic compromise who may require thrombolytic therapy, mechanical aspiration or MCS devices.

### 5.3. Oxygenation and Ventilation

High-flow nasal cannula or initiation of mechanical ventilation may be warranted for the treatment of hypoxemia, and caution should be taken during the induction of anesthesia, intubation, and positive-pressure ventilation. In particular, positive intrathoracic pressure induced by mechanical ventilation may reduce venous return and worsen low cardiac output due to RV failure in patients with high-risk PE; therefore, positive end-expiratory pressure should be applied cautiously [4]. 

### 5.4. Vasoactive Medications

Hemodynamic support with vasopressors should be considered early. When the central venous pressure is low, a conservative fluid challenge could be considered, as it has the potential to enhance the cardiac index among individuals [22]. However, volume loading might cause RV over distention and subsequently result in a decrease in systemic cardiac output (CO) [23]. Studies conducted in experimental settings indicate that pursuing aggressive volume expansion does not seem to offer advantages and might even deteriorate RV function [24]. 

Norepinephrine is the most commonly used vasoactive agent [24]. Norepinephrine can augment cardiac output and systemic vascular resistance and may be used independently or in combination with other agents. Dobutamine and milrinone act as inotropes and improve cardiac output. Dobutamine does not significantly impact pulmonary arterial pressure; however, milrinone can reduce pulmonary arterial pressure and reduce pulmonary vascular resistance [25]. Studies in animal models have explored isoproterenol, amrinone, and milrinone, but these agents should not be primarily used in hypotensive patients [26,27]. Vasoactive agents should be used primarily to restore hemodynamic compromise prior to utilizing MCS devices, given the complications and technical expertise these devices take to place and manage.

Should cardiac arrest occur, advanced life support guidelines should be followed [28]. Thrombolytic therapy should be considered, and given first over anticoagulation. If thrombolytic medication is administered, cardiopulmonary resuscitation should be maintained until resuscitative efforts are deemed to be futile [5,21,29].

### 5.5. Thrombolysis

Thrombolysis is recommended as an initial approach in high-risk PE, absent contraindications. A meta-analysis of five randomized controlled trials that included patients with massive PE demonstrated that systemic thrombolytic treatment reduces the risk of mortality or recurrent PE by 55% [30]. However, thrombolytic therapy may be delivered via various approaches, including systemic IV infusion, catheter-directed low-dose, and pharmacomechanical, each with its own advantages and disadvantages regarding cost, invasiveness, and bleeding risk. Systemic thrombolysis poses a significant risk of bleeding, with a 20% likelihood of major bleeding and a 2% risk of intracranial hemorrhage [29,31,32].

Catheter-directed thrombolysis may be comparable or superior to systemic thrombolysis with the added benefit of reducing the risk of major and intracranial bleeding [5]. This approach is less likely to cause major bleeding because a lower cumulative dose of the thrombolytic agent is delivered directly into the clot via a multi-side hole infusion catheter. An advanced alternative to conventional catheter-directed thrombolysis is ultrasound-assisted thrombolysis, which employs a dual-lumen catheter for potentially enhanced thrombolysis. A randomized controlled trial showed that compared with conventional heparin treatment, ultrasound-assisted thrombolysis in patients with intermediate-risk PE and echocardiographic RV to left ventricular dimension (RV/LV) ratio ≥1.0 was associated with a greater reduction in RV/LV diameter ratio at 24 h without an attendant increase in bleeding. At the 90-day mark, the difference in RV/LV reduction was no longer statistically significant [33]. The primary theoretical advantage of ultrasound-assisted thrombolysis over standard catheter-directed thrombolysis is its potential for more efficient, targeted delivery of the thrombolytic agent within a shorter time frame [5]. The forthcoming results of the Higher-Risk Pulmonary Embolism Thrombolysis (HI-PEITHO)trial (ClinicalTrials.gov Identifier: NCT04790370) aim to evaluate the effectiveness of combining ultrasound-assisted, catheter-directed thrombolysis with anticoagulation therapy versus using anticoagulation therapy alone in treating patients with acute, intermediate-high risk PE [34].

### 5.6. Percutaneous Pulmonary Embolectomy

Various techniques and specialty catheters are available to perform percutaneous pulmonary embolectomy. These include maceration via a rotation of a pigtail catheter, manual aspiration through a large-bore sheath or catheter, and other methods such as rheolytic thrombectomy. Clinical studies, such as the FLAME trial, suggest that mechanical thrombectomy is feasible, safe and potentially effective for patients with acute high-risk PE [35]. Also, the FLASH trial underscores the effectiveness and safety of mechanical thrombectomy using the FlowTriever System in patients with intermediate to high-risk PE, demonstrating significant haemodynamic improvements and low mortality rates [36].

Although systemic thrombolysis is generally the initial treatment of choice for patients with high-risk PE, the Management Strategies and Prognosis in Patients with Pulmonary Embolism (MAPPET) registry revealed that 40% (193 out of 478) of patients receiving fibrinolysis had at least one relative contraindication and approximately one-third of patients had absolute contraindications to this therapy [25,37]. Furthermore, in the International Cooperative Pulmonary Embolism Registry (ICOPER) cohort of 304 patients treated with fibrinolysis, 21.7% experienced major bleeding complications, and 3.0% encountered intracranial bleeding [38]. Given these limitations, surgical embolectomy is another option. Still, this approach incurs a high risk of morbidity and mortality, particularly in patients who have had unsuccessful thrombolysis, and this technique is offered only in specialized medical centers. Therefore, percutaneous pulmonary embolectomy is increasingly recommended for patients with high-risk PE when technical expertise is available [39].

### 5.7. Surgical Embolectomy

Surgical embolectomy should be considered when fibrinolytic treatment is either contraindicated or has not led to improved hemodynamics. Despite the need for cardiopulmonary bypass and the administration of high-dose heparin during surgical embolectomy, this procedure is associated with a lower incidence of major bleeding when compared to systemic fibrinolysis therapy [40,41]. Recent non-randomized studies have reported favorable surgical outcomes in patients with intermediate and high-risk PE [42,43,44]. Extracorporeal membrane oxygenation (ECMO) when combined with surgical embolectomy may be beneficial, especially in patients with high-risk PE, even in situations that require cardiopulmonary resuscitation [45].

## 6. Mechanical Circulatory Support

**Figure 2:**
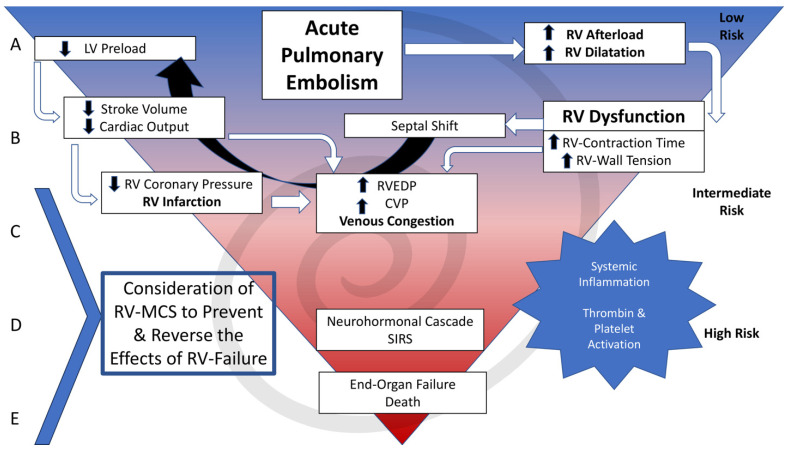
Cascading event occurring after pulmonary embolism. CVP, central venous pressure; LV, left ventricular; RV, right ventricular; RV-MCS, right ventricular mechanical circulatory support; RVEDP, right ventricular end-diastolic pressure; SIRS; Systemic Inflammatory Response Syndrome.

**Figure 3:**
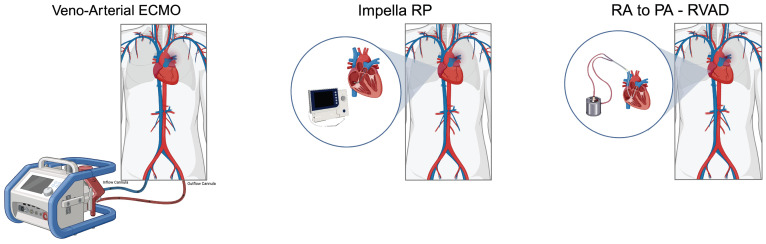
Right ventricular mechanical circulatory support devices can be used in high-risk pulmonary embolism. (Created with BioRender.com).

Right ventricular mechanical circulatory support (RV-MCS) is utilized in patients with refractory shock to prevent or reverse shock in patients with symptoms refractory to initial medical therapies listed above, Figure 2. The primary goal of RV-MCS technologies for to address right ventricular failure (RVF). Options include VA-ECMO and isolated right ventricular assist devices (RVAD) see Figure 3. Percutaneous RVADs include Impella RP/Flex (Abiomed, Danvers, MA) and Protek Duo (CardiacAssist Inc., Pittsburgh, PA, USA). Surgical RVAD includes devices such as Centrimag (Abbott Vascular, Green Oaks, IL, USA), see Table 1. 

**Figure 4:**
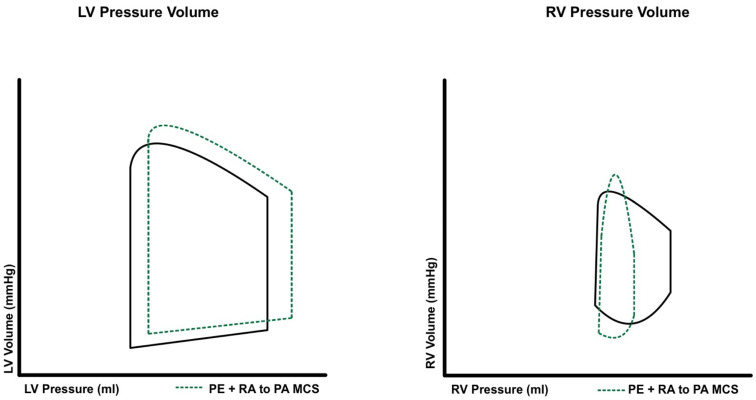
Pressure–volume changes that occur to the right and left ventricular with the use of right ventricular assist devices in high-risk pulmonary embolism.

**Figure 5:**
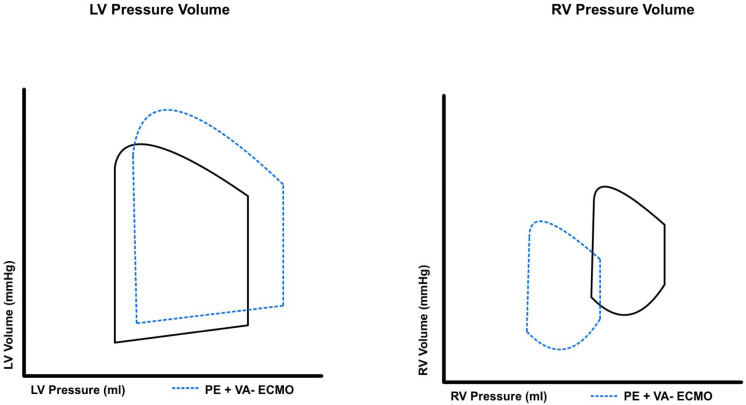
Pressure–volume changes that occur to the right and left ventricular with the use of extracorporeal membrane oxygenation in high-risk pulmonary embolism.

RV-MCS devices are classified by their operative mechanism and are either direct RV bypass or indirect RV bypass systems. Select RV-MCS devices allow for not only hemodynamic support but also the ability to provide oxygenation in patients with severe PE. RVADs facilitate blood flow from the right atrium to the pulmonary artery, thus providing direct RV bypass. RVAD decreases central venous pressure, mildly increases pulmonary artery pressure and improves overall native cardiac output, see Figure 4. On the other hand, venoarterial ECMO (VA-ECMO) bypasses blood from the right atrium to the femoral artery, offering an indirect RV bypass. ECMO thus decreases central venous pressure and reduces pulmonary artery pressure, see Figure 5.

When treating patients with high-risk PE who are experiencing hemodynamic collapse, the use of temporary MCS devices plays a pivotal and may be lifesaving [46], as recent guidelines from the ESC reserve MCS for patients with acute PE who have circulatory collapse or cardiac arrest [4]. Due to the scarcity of literature, there are no established selection criteria for the use of MCS devices in patients with high-risk PE. A study by the Massachusetts General Hospital PERT team, spanning nine years, documented ECMO deployment in 13 instances. The application of ECMO was notably selective, engaging primarily in situations of cardiac arrest prior to support initiation [47] In contrast, some centers adopt an early intervention strategy, incorporating ECMO for all identified high-risk or massive PE patients from the outset. This approach was highlighted in research by Pasrija et al., evaluating 19 patients over two years, with only five experiencing cardiac arrest before ECMO support [48]. MCS in high-risk PE should be considered with worsening hemodynamics including lower blood pressure, rising central venous pressure, decreasing pulse pressure, worsening oxygenation and clinical or laboratory signs of end-organ damage (including elevated lactate levels).

### 6.1. Impella RP 

The Impella RP is a microaxial-flow pump that features a 22F impeller affixed to an 11F catheter for facilitating blood flow from the right atrium to the pulmonary artery, see Figure 3. The Impella RP is inserted into the pulmonary artery with a 23F venous peel-away sheath and a 0.018 in-wire serving as a monorail system, which requires a single venous access point, typically the right femoral vein. The Impella RP Flex is a similar, but newer system, which can be delivered using a femoral or internal jugular approach. After positioning the pump, the 23F sheath is exchanged with a staged 11F to 23F repositioning sheath. during sheath removal, hemostasis may be achieved via manual pressure and either a purse string or a deep mattress suture. The Impella RP cannot oxygenate the blood [49]. Although the Impella device is effective for treating RV infarction, consistent evidence to support its role in treating high-risk PE is lacking. In an independent study involving five patients who received Impella RP for shock due to high-risk or intermediate high-riskPE, treatment was associated with a significant improvement in cardiac index and hemodynamics, with no adverse impact on renal function, hemoglobin, and platelet levels except one patient’s experienced a decline in hemoglobin levels without needing a transfusion. All five patients survived to discharge [19]. 

Another approach for treating right ventricular failure from PE is the insertion of an extracorporeal centrifugal-flow pump in conjunction with a 29 Fr or 31 Fr single-access dual-lumen cannula, which can be inserted percutaneously through the internal jugular vein, Figure 3. This system facilitates the transfer of blood from the right atrium to the main pulmonary artery, which can generate a blood flow of 4–5 L per minute [50]. Additionally, the device can also be connected to an oxygenator, providing reversal of hypoxia and reducing the associated vasoconstriction and elevated peripheral vascular resistance (PVR). European Medicines Agency has approved the LifeSPARC Pump and ProtekDuo cannula for 30-day support and the FDA for 6-day support [51,52].

### 6.2. Surgical RA to PA RVAD

In addition to percutaneous methods, surgically implanted RVADs provide another option for mechanical RV support, but without the option of O_2_ supplementation. The surgical procedure additionally requires median sternotomy [53]. Such devices are often employed post-LVAD implantation, supporting patients through severe RV failure [54,55,56]. The CentriMag system can produce flows up to 10 L/min and sustain support for 30 days, with minimal thromboembolic risks [54].

### 6.3. Data Regarding RVADs

In one systematic review of 17 patients, temporary RVADs, including eight Impella RP and nine other RVAD variations (two percutaneous and seven central), were associated with a survival rate of 94% [57]. A case study reported a patient who fully recovered from severe cardiogenic shock secondary to high-risk PE after being treated with the ProtekDuo for RV support [58]. A case series of four patients treated with the Protek Duo with an oxygenator demonstrated improved hemodynamic status and RV function with no device-related complications. Patients were decannulated after an average of 8 days, although one patient died of hospital-acquired pneumonia 15 days after decannulation. These findings highlight the potential that such RV-MCS devices may have for patients with PE, but more comprehensive and robust evidence is needed to draw definitive conclusions [59].

### 6.4. ECMO

ECMO is the most commonly used MCS in patients with high-risk PE. Peripheral VA-ECMO is typically placed using a 15–19F arterial access and 21–29F venous access, bypassing the RV and displacing 4–6 L/min of blood from the right atrium to the iliofemoral arterial system and O_2_ supplementation, see Figure 3. VA-ECMO is one of the most reliable and rapid ways to decrease RV overload, improve RV function and hemodynamics, and restore tissue oxygenation [15]. The device can be used as an initial stand-alone therapy or with the use of thrombolytic therapy in massive PE patients. Prior data showed high mortality rates in patients already having high mortality, but several more recent retrospective studies suggest that VA-ECMO may have high efficacy. A Japanese Diagnosis Procedure Combination database study revealed an increasing use of ECMO for treating PE, from 11.0% to 21.3% between 2010 and 202 [60]. However, the merits of ECMO relative to other therapies for treating PE are still unclear. VA-ECMO is usually deployed for PE within cardiogenic shock, contraindication to thrombolysis, or unsuccessful reperfusion therapy [61]. It can also be utilized as a standalone therapy when paired solely with anticoagulants [15]. Alternatively, it may be used as a bridge to reperfusion therapies, most commonly surgical or percutaneous embolectomy [17]. Using ECMO before surgery can yield different outcomes compared to either post-surgical ECMO use or standalone ECMO treatment. In a study of 52 cases, the mortality rate was 78% in patients treated with ECMO alone, versus 29% in those treated with ECMO and surgical embolectomy. It is worth noting that these results may be skewed by selection bias, as most patients not chosen for surgery were already considered to have a poor prognosis [16]. 

In a large retrospective analysis that included over one and a half million patients hospitalized with PE, more than 2000 were treated with ECMO, and patients treated with VA-ECMO alone or in combination with reperfusion therapy had significantly lower in-hospital mortality than patients treated with thrombolysis [62]. However, a meta-analysis of 24 observational studies that included 1947 patients with PE concluded that evidence is lacking to show that VA-ECMO improves the short-term survival of patients relative to control patients [63]. Nevertheless, patient populations and study parameters differ widely across studies, and more high-quality data are needed to determine whether ECMO favorably impacts mortality or outcomes of patients with PE. ECMO support is typically required for 5-10 days, with a maximum implant time of 3–4 weeks [64]. 

## 7. MCS Device Selection

Choosing the optimal MCS strategy for patients should also be tailored to patient hemodynamics, availability of other therapeutic interventions, as well as operator and institutional experience, Table 1 [57]. 

RVAD technologies bypass the right ventricle, and because they deliver blood to the pulmonary artery, increase pulmonary vascular resistance. There is a theoretical risk of pulmonary hemorrhage due to active flow from the RVAD; this is particularly concerning in patients who have been treated with thrombolytics. However, the main advantage of RVAD is that only venous access is needed. Avoiding surgical interventions or large-bore arterial access may be advantageous in select patients at high risk for bleeding. A large retrospective analysis comparing RVAD to VA-ECMO investigators observed more favorable outcomes for patients treated with an RVAD; however, these patients had normal LV function and relatively fewer co-morbidities [65]. Further study on RVAD is warranted. 

At present, the most commonly used RV-MCS device in patients with high-risk PE is VA-ECMO. VA-ECMO bypasses the pulmonary circulation and avoids increasing flow in the pulmonary artery. It allows for oxygenating the patient [66]. VA-ECMO can also be utilized outside of the operating room and cath lab and, therefore, may be the most accessible device to deliver in a timely manner at sites with ECMO teams. The major disadvantage of VA-ECMO is the need for large-bore arterial access, which can increase the risk of bleeding and limb ischemia. 

When choosing to utilize MCS is it important to keep patients’ underlying co-morbidities and shock phenotype in mind. For example, patients may present with acute PE in the setting of chronic pulmonary arterial hypertension or pre-existing right ventricular dysfunction, in such patients temporary MCS may help the acute hemodynamic changes; however, recovery may be impacted by the pre-existing disease state. Similarly, patients presenting with acute PE in the setting of pre-existing left ventricular failure may present with significant hemodynamic collapse. It would be important to consider biventricular MCS devices such as VA-ECMO in such patients. In any patient with pre-existing ventricular dysfunction, careful consideration of goals of care should occur and with care decisions guided by a multi-disciplinary care team.

## 8. Weaning MCS 

Weaning from MCS devices involves complex and critical timing within a clinical context [67,68,69,70]. If the RV function fails to adequately recover, patients are likely to spiral into a state of shock [67,71]. Weaning is generally initiated through a series of trials by incrementally decreasing device support while monitoring hemodynamic and respiratory stability. Specific clinical parameters, such as blood pressure and pulse pressure, invasive hemodynamics, such as central venous pressure (CVP), pulmonary artery pulsatility index (PAPi), cardiac output (CO)/cardiac index (CI), perfusion parameters (lactate, urine output, etc.), and dependency on vasoactive agents should inform the decision to wean patients from MCS. During weaning from MCS, respiratory support via invasive or non-invasive mechanical ventilation requires careful monitoring and titration, since MCS may also provide oxygenation. Upon verifying hemodynamic stability, device output should initially be decreased slowly, following the associated parameters carefully. Patients who sustain stability with limited mechanical assistance for multiple hours, are ready for device removal. 

When weaning RVADs or VA-ECMO we suggest progressive reductions by 0.5–1.0 L/min every 2–3 h or slower depending on the patient’s baseline hemodynamics. We also suggest utilizing both echocardiographic and hemodynamic assessments to guide the process [72]. Parameters to follow include central venous pressure (ideally maintained < 15 mmHg) and pulmonary capillary wedge pressure maintained below 24 mm Hg [73] Current literature does not establish definitive PAPi cutoffs for weaning from ECMO in the case of pulmonary embolism. While PAPi values below 1.0 have been linked to right ventricular failure in acute myocardial infarction, and values below 1.85 indicate RV failure after LVAD implantation, these parameters are not directly transferable to ECMO weaning.

A detailed echocardiographic evaluation of ventricular performance has been shown to be highly predictive of successful cardiac recovery. Specifically, for the right ventricle, metrics such as ejection fraction, fractional area change, and strain, offer prognostics for successful weaning when compared to tricuspid annular plane systolic excursion or tricuspid regurgitation severity [67,73,74,75,76]. A study explored the echocardiographic indicators of RV function that predict the success of weaning. It revealed that three-dimensional RV ejection fraction (EF) was closely linked to successful weaning outcomes, with a threshold value of about 25% [67,77]. Other echocardiographic parameters to consider include tricuspid annular plane systolic excursion (TAPSE), S’ and fractional area change (FAC). It is important to note that while TAPSE of less than 17 mm is associated with RV dysfunction, there is no specific cutoff determined for ECMO weaning. However, some studies recommend weaning algorithms based on cumulative clinical experience [78]. Generally, the focus is on assessing RV recovery and involves a gradual reduction in pump flow, to a specific percentage of the patient’s optimal flow over a defined period. No additional specific parameters have been analyzed for evaluating RV function throughout a weaning trial and while specific echocardiographic protocols for ECMO weaning have not been established, a methodology akin to that used for weaning from a VAD could be applied.

## 9. Mitigating MCS Complications

Major complications associated with MCS include bleeding, thromboembolism, neurological injury, cannulation-related injury and ischemia [64]. Bleeding is the most common complication of MCS, resulting from the need for therapeutic anticoagulation treatment over an extended period of time. Additionally, platelet damage can lead to thrombocytopenia compounding this risk. Cannulation-related injuries include hemorrhage, dissection, limb ischemia (from arterial thromboembolism or vessel occlusion from the large bore device when a reperfusion sheath is not used with VA ECMO), and resultant compartment syndrome [79]. Ongoing changes in the design of RVAD and ECMO devices aim to decrease these complications. For example, modern heparin-coated ECMO circuits allow anticoagulation therapy to be discontinued for a limited period in the case of active bleeding. Importantly, regular and careful inspection of tubing and monitoring of gradients across the oxygenator is recommended to detect thrombi that can form in the circuit [80]. Oxygenated blood returning from the ECMO circuit to the aorta can also result in north–south syndrome (Harlequin Syndrome) which arises when the interaction between retrograde blood flow from peripheral VA-ECMO (well oxygenated) and antegrade flow from the heart (usually poorly oxygenated) creates a “watershed” zone within the aorta distal to the left subclavian artery, leading to a mismatch in oxygenation. This condition is characterized by lower body regions receiving well-oxygenated blood from the ECMO, while the upper body, including the brain, is supplied with less oxygenated blood, resulting in differential cyanosis and the distinctive appearance akin to a Harlequin [81] and must be monitored for [74]. 

## 10. Conclusions

The realm of interventional treatment for PE is evolving, and MCS devices offer promise for treating patients with high-risk PE. However, proper use of these devices is evolving, and clinical guidelines recommend their use primarily in acute, life-threatening scenarios. The choice of specific MCS devices must be individualized to a patient’s condition and risk profile. Although data on long-term outcomes and quality of life post-MCS for PE are limited, ongoing advancements suggest a hopeful trajectory for patient survival and recovery, underscoring the need for comprehensive follow-up care and rehabilitation protocols. While early results are encouraging, more robust, randomized controlled trials are needed to establish the effectiveness and safety of MCS devices for treating patients with PE.

## Figures and Tables

**Table 1:** Types and characteristics of right ventricular mechanical circulatory support devices.

	Impella RP	Percutaneous RA to PA RVAD	Surgical RA to PA RVAD	VA-ECMO
Flow (L/min)	~4.0–5.0 L/min	~4–5 L/min	~4–7 L/min	~4–6 L/min
Mechanism of action	Transvalvular micro-axial pump	Centrifugal cardiac bypass	Centrifugal cardiac bypass	Centrifugal cardiopulmonary bypass
Access site	Femoral or Jugular Vein	Jugular Vein	Surgical RA and PA access	Femoral/Axillary Artery Femoral/Jugular Vein
Sheath size	23F	29 Fr 31 Fr	Drainage 32–36 Fr Return 32–36 Fr	Drainage—21–29 Fr Return—15–19 Fr
Pre-capillary PA Flow	↑	↑	↑	↓
Advantages	Venous only access	Venous only access Ability to oxygenate	Ability to oxygenate	Biventricular support, Ability to oxygenation
Disadvantages	Unable to oxygenate	Risk of SVC Syndrome	Surgical access required	Requires large bore arterial access, Increased afterload can affect LV function

LV, left ventricular; PA, pulmonary artery; RA, right atrial; RVAD, right ventricular; SVC, superior vena cava; VA-ECMO, venoarterial extracorporeal membrane oxygenation; ↑, increase; ↓, decrease.

## Data Availability

Data is unavailable.

## Abbreviations

AHA	American Heart Association
aPTT	activated partial thromboplastin time
CI	cardiac index
CO	cardiac output
CVP	Central venous pressure
DVT	deep vein thrombosis
ECMO	extracorporeal membrane oxygenation
ESC	European Society of Cardiology
ICOPER	the International Cooperative Pulmonary Embolism Registry
IV	intravenous
LV	left ventricular
MAPPET	Management Strategies and Prognosis in Patients with Pulmonary Embolism registry
MCS	mechanical circulatory support
PA	pulmonary artery
PAPi	the pulmonary artery pulsatility index
PE	pulmonary embolism
PERT	pulmonary embolism response team
PVR	peripheral vascular resistance
pRVAD	percutaneous right ventricular assist device
RA	right atrium
RV	right ventricular
RVAD	right ventricular assist device
RV-MCS	right ventricular mechanical circulatory support
VA-ECMO	venoarterial extracorporeal membrane oxygenation
